# Tooth Powders

**Published:** 1887-08

**Authors:** 


### TOOTH POWDERS.

*The National Druggist* says that some time since it was discovered that the addition of powdered soapstone to the water in steam boilers prevented the deposit of lime concretions. Straightway some brilliant genius was possessed of the idea that, as the tartar upon the teeth was of the same general character with the incrustation of steam boilers, all that was necessary to prevent its deposition was to add soapstone to a dentifrice, and the following formula was devised and recommended by leading medical journals:

| | |
|---|---|
| Powdered steatite, | ℥ xv. |
| " alum (or cream of tartar), | ℥ iss. |
| " cochineal, | ℥ iiss. |
| Essence peppermint, | gtt. xx. |

A more absurd preparation for the purposes desired could scarcely be conceived. In the first place, soapstone is a lubricator, and its use in a tooth powder will not furnish sufficient friction to properly clean the teeth.

There are few substances more destructive to the tooth tissues than is alum, the double salt of alumina and potash, hence its use in a tooth powder is entirely without excuse.

The addition of two and one-half drachms of cochineal is too absurd for comment, while the peppermint as a flavor will not serve to cover the taste of the other ingredients nearly as well as would some other material. We can only repeat what we have before said—that in the preparation of tooth powders it is better to leave the whole to some one who makes a specialty of the business; one who has all the facilities for a perfect trituration and mixing of the ingredients, and who prepares the dentifrice from a standard and approved formula.

